# Simulation Analysis of Improving Microfluidic Heterogeneous Immunoassay Using Induced Charge Electroosmosis on a Floating Gate

**DOI:** 10.3390/mi8070212

**Published:** 2017-07-04

**Authors:** Qingming Hu, Yukun Ren, Weiyu Liu, Ye Tao, Hongyuan Jiang

**Affiliations:** 1 School of Mechatronics Engineering, Harbin Institute of Technology, West Da-zhi Street 92, Harbin 150001, Heilongjiang, China; qminghu@gmail.com (Q.H.); liuweiyu@chd.edu.cn (W.L.); tarahit@gmail.com (Y.T.); 2 School of Mechatronics Engineering, Qiqihar University, Wenhua Street 42, Qiqihar 161006, Heilongjiang, China; 3 State Key Laboratory of Robotics and System, Harbin Institute of Technology, West Da-zhi Street 92, Harbin 150001, Heilongjiang, China; 4 School of Electronics and Control Engineering, Chang’an University, Middle-section of Nan’erHuan Road, Xi’an 710064, Shaanxi, China

**Keywords:** heterogeneous immunoassay, induce charge electroosmosis, binding efficiency

## Abstract

On-chip immuno-sensors are a hot topic in the microfluidic community, which is usually limited by slow diffusion-dominated transport of analytes in confined microchannels. Specifically, the antigen-antibody binding reaction at a functionalized area cannot be provided with enough antigen source near the reaction surface, since a small diffusion flux cannot match with the quick rate of surface reaction, which influences the response time and sensitivity of on-chip heterogeneous immunoassay. In this work, we propose a method to enhance the transportation of biomolecules to the surface of an antibody-immobilized electrode with induce charge electroosmotic (ICEO) convection in a low concentration suspension, so as to improve the binding efficiency of microfluidic heterogeneous immunoassays. The circular stirring fluid motion of ICEO on the surface of a floating gate electrode at the channel bottom accelerates the transport of freely suspended antigen towards the wall-immobilized antibodies. We investigate the dependence of binding efficiency on voltage magnitude and field frequency of the applied alternate current (AC) electrical field. The binding rate yields a factor of 5.4 higher binding for an applied voltage of 4 V at 10 Hz when the Damkohler number is 1000. The proposed microfluidic immuno-sensor technology of a simple electrode structure using ICEO convective fluid flow around floating conductors could offer exciting opportunities for diffusion-limited on-chip bio-microfluidic sensors.

## 1. Introduction

Immunoassays, which refer to the specific binding interaction between the free antigens in the analytes and the immobilized antibody, have been widely applied in point-of-care detections, biological research, quality control, pharmaceutical research, and environmental diagnostics for their high selectivity and sensitivity. Conventional immunoassays, such as microarrays [1] or enzyme-linked immune sorbent assays (ELISA) [2], require larger sample volume and longer incubation time due to their labor-intensive and sophisticated fluid handling steps at various stages of the assay. The most commonly used label-free bio-molecule detection methods, such as electrochemical impedance spectroscopy (EIS) [3], surface plasmon resonance (SPR) [4], and quartz crystal microbalance (QCM) [5] also have been utilized to detect specific proteins and bacteria. However, the dependence on the surface-binding reaction, which is limited by slow reaction rates and long detection time, restrict the versatile application in some circumstances. In addition, the limited throughput restrains the time-critical immunoassay application. With advancement in microfabrication and the miniaturization of the microfluidic systems, integrating the biosensors into lab-on-a-chip systems has received considerable attention [6]. Compared with the conventional immunoassays method, microfluidic immuno-sensors offer tremendous advantages to deliver the target antigen onto the transducer surface in a continuous-flowing manner employing small samples and including high throughout, small volume, short analysis time, and high sensitivity. The mass transport process where the specific analyte is transported from the bulk solution towards the receptor surface is influenced by the diffusion limitations due to the laminar flow in the microchannel. Therefore, the microfluidic immunoassays are still limited in response time and sensitivity due to the slow diffusion of target molecules/proteins to the detection surface, which limits the overall performance of the immuno-sensors requiring the fast surface reactions.

Various microfluidic mechanisms have been employed to manipulate the flow pattern in microchannel for bio-sample diagnosis, such as, hydrodynamic pressure [7,8], electric forces (electrohydrodynamics EHD) [9,10,11,12,13,14,15,16,17], magnetic forces [18], and optical forces [19,20]. For example, Marwa [7] numerically simulated the confinement effect on the binding reaction using a microchannel-based flow confinement method with a perpendicular makeup flow to confine analyte transport toward the reaction surface. As an alternative, the alternate current electrokinetics (ACEK) has gained prevalence to actuate fluid motion and manipulate particles to the desired location with the low voltage applied [21,22,23]; this technique mainly includes dielectrophoresis (DEP) [24,25,26], AC electroosmosis (ACEO) [27,28,29,30], and AC electrothermal (ACET) [31,32,33,34,35]. Yang [22] presents an overview of microfluidic technologies to enhance concentration sensitivity and detection resolution in microfluidic devices. Hart [10] proposed a microfluidic-based sensor to enhance the transport of analyte to a transducer surface to improve the detection time and sensitivity of immunoassays using ACEO. Arising from the movement of induced charges from the local variations in fluid conductivity and permittivity due to Joule heating, AC-driven electrothermalmicro-stirring was exploited to enhance the binding and reaction between specific antigen and antibodies in microfluidic immuno-sensors numerically and experimentally [36,37]. The characteristic of high electrolyte conductivity used in ACET and the generated Joule heat sometimes hinders its application in immunoassay systems.

The induced-charge electrokinetics (ICEK) phenomena has emerged as a potential method for fluid manipulation and received considerable attention from the microfluidic community. Similar to ACEO, arising from the interaction of the applied electrical field with its own induced diffuse charge in a thin boundary layer on a polarizable surface in contact with an electrolyte, the induced-charge electroosmosis (ICEO) flow at the solid/electrolyte interface has been widely used for pumping [38,39], mixing [40,41,42,43], and particle trapping [44,45,46,47,48,49]. The introduction of a floating gate electrode has the advantage of flexible configuration, and it is free from the external circuit, making it convenient for device integration. Recently, we exploited the ICEO vortex to trap micro-sized particles and realize the scaled particle by focusing on the surface of polarizable electrode strips with low voltage alternating current (AC) electrical fields [35,47,48,50,51,52]. Pascall and Squires [53] presented an automated experimental platform to study the ICEK slip velocity over electrodes under a variety of conditions and demonstrated that both the presence of a dielectric coating and an equivalent ion adsorption capacitance on the electrode surface was accountable for the larger flow velocity predicted from the standard theory than that obtained from actual experimental observations.

Therefore, when the reaction rate is much faster than the transport of reactants to the transducer surface in microfluidic devices, it is essential to develop a label-free and highly sensitive detection technology to improve the diffusion rate and increase the antibody-antigen binding efficiency for rapid detection of biomolecules relying on diffusion dominated transport of analytes. In this paper, we demonstrate a method to deliver the target antigen to the detection surface under low analyte concentrations (typically < 0.05 S/m, to avoid double layer shrinkage at higher conductivities) and enhance the performance of heterogeneous immunoassays using ICEO convective flow on polarizable surfaces. The generated circular micro-stirring fluid motion near the floating electrode surface enhances the transportation of antigens towards immobilized ligands and reduces the thickness of the diffusion boundary layer, significantly increasing the binding efficiency compared to the conventional pressure driven flow system. The proposed lab-chip immuno-sensor system has the merit of portability, rapid detection, and low energy consumption.

## 2. Theory and Methods

### 2.1. Device Design

**Figure 1:**
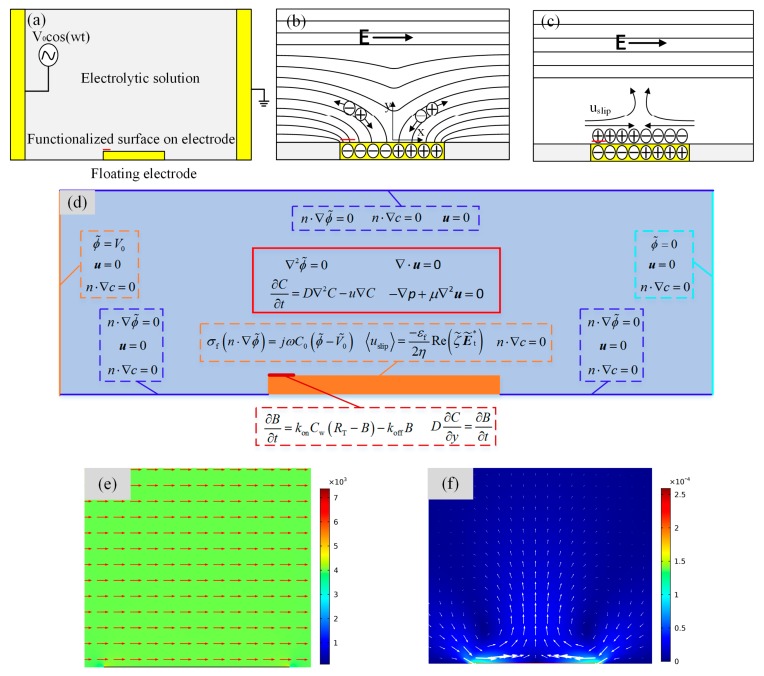
(**a**) Sketch of the microfluidic device geometry and electrode placement used to enhance the immunoassays in our experiment; (**b**,**c**) Schematic illustrations of the induced double layer and induce charge electroosmotic (ICEO) slip formation around the floating metal strip: (**b**) the counter-ionic electro-migration to the bipolar electrode following the normal field component when a sudden electric field is applied; (**c**) the formation of the dipolar diffuse screening cloud at the metal/electrolyte interface after a characteristic resistance–capacitance (RC) charging time, giving rise to the ICEO streaming flow; (**d**) the computational domain of the COMSOL simulation showing the governing equations (solid line box) and the boundary condition (dotted box); (**e**) an arrow and surface plot of electric field phasor amplitude at *V*_1_ = 4 V and 10 Hz in the vicinity of the gate terminal (unit: V/m); (**f**) Transverse flow field of ICEO on the gate electrode with an applied voltage magnitude of 4 V and frequency of 10 Hz along the axial channel of 1000 μm in length.

To explore the ICEO effect on the two-dimensional fluid on the immuno-sensor, we designed the microfluidic chip, as shown in Figure 1a. A floating electrode with a width of 50 μm and height of 200 nm was deposited on the bottom of the microchannel with the height and length of 200 μm and 1000 μm, respectively. For a suddenly applied electrical field, an ionic conduction current, normal to the metal strip surface, injected bipolar ions into a thin double layer at the metal/electrolyte interface under the action of the original normal field component on the ideally polarizable metal surface (Figure 1b). After a characteristic resistance–capacitance (RC) charging time $\tau_{\mathrm{RC}}=RC_{D}/\sigma_{f}\left(1+\delta \right)=\varepsilon_{f}R/\sigma_{f}\lambda_{D}\left(1+\delta \right)$ (where, *R* and $\lambda_{D}$ are the characteristic macroscopic length scale and the Debye thickness of the induced double layer (IDL), respectively), the bulk field lines outside the diffuse layer were fully expelled and the ionic charge clouds were formed on the metal strip surface. This makes the conducting surface behave like an insulator, resulting in a pair of counter-rotating ICEO micro-vortices above the inducing electrode surface due to the action of tangential electrical field components on the induced free charge within the IDL (as shown in Figure 1c). The generated micro-eddies on the surface of the bipolar metal strip may be exploited for improving antigen-antibody bound reactions on the functionalized part of the gate terminal (denoted by the red line segment in Figure 1a–c) in immunoassays.

### 2.2. Induced Double-Layer Charging at the Solid/Electrolyte Interfance and ICEO Flow

To illustrate the transient ICEO for analytical convenience, the complex phasor amplitude of the electric field variable as denoted by a tilde is introduced, e.g., ϕ(t)=Acos(ωt+θ)=Re(Aejθejωt)=Re(ϕ˜ejωt), where, Re (…) is the real part of (…). *A*, *w*, and *ɵ* are the amplitude, angular frequency, and phase angle of the electrostatic voltage signal, respectively.

In the thin electric double layer limit, the ohmic current from the bulk resistance charges the IDL like a capacitor skin. Hence, the normal conducting current arriving at the outer rim of the Debye layer can be obtained [47,48]:

(1) $$n\cdot \tilde{J}=-\sigma_{f}\left(n\cdot \nabla \tilde{\phi }\right)=j\omega \frac{C_{D}}{1+\delta }\left({\tilde{V}}_{0}-\tilde{\phi }\right)$$

where ***n*** is the unit normal vector, pointing from the electrode into the bulk. ϕ and $\sigma_{f}$ are the bulk potential and the liquid conductivity, respectively. $\delta =\frac{C_{D}}{C_{S}}$ denotes the surface physical capacitance ratio of the diffuse layer *C*_D_ to the stern layer *C*_S_. ${\tilde{V}}_{0}$ is the fixed potential phasor of the central floating electrode as obtained by charge conservation.

When the left electrode is activated by AC sinusoidal voltage $V_{0}\cos\left(\omega t\right)$, the right electrode is grounded, phasor amplitude of the induced zeta potential contributing to induced electrokinetic flows is $\tilde{\zeta }={\tilde{\phi }}_{\mathrm{OHP}}-\tilde{\phi }=\frac{1}{1+\delta }\left({\tilde{V}}_{0}-\tilde{\phi }\right)$, here, ${\tilde{\phi }}_{\mathrm{OHP}}$ is the transient potential at the outer Helmholtz plane between the diffuse layer and the stern layer.

The expression for the time-averaged nonlinear electrokinetic slip in AC oscillation is derived from the generalization of the Helmholtz-Smoluchowski formula, which is then inserted into the Stokes equation as an effective boundary condition on the polarizable surface of the floating electrode [44].

(2) 〈uslip〉=−εfη〈ζEt〉=−εfη12Re(ζ˜E˜t*)=−εfη12Re((V˜0−ϕ˜)1+δE˜t*)=12εfη11+δRe((ϕ˜−V˜0)(E˜−E˜⋅n⋅n))

where $\varepsilon_{f}$ and η are the permittivity and dynamic viscosity of the liquid, respectively, 〈…〉 is the time-averaged value in an alternating filed, and the asterisk symbol * indicates the complex conjugate operator.

### 2.3. Antigen Reation and Bound Antigen Enhancement

The antigen transport motion to and from the reaction surface within the microchannel can be described with the convective scalar equation based on the Fick’s second law:

(3) $$\frac{\partial C}{\partial t}=D\nabla^{2}C-u\nabla C$$

where, *C* denotes the bulk concentration of the antigen, and *u* and *D* are the fluid velocity and diffusion coefficient of the antigen suspended in the fluid, respectively. An initial antigen concentration of *C*_0_ = 0.1 nM is introduced into the microchannel cavity.

The binding reaction between the antigen in electrolyte and the immobilized antibodies at the functionalized surface can be assumed to follow the first-order Langmuir absorption model [54,55]:

(4) $$\frac{\partial B}{\partial t}=k_{\mathrm{on}}C_{w}\left(R_{T}-B\right)-k_{\mathrm{off}}B$$

where, *B* is the surface concentration of the bound antigen along the reaction surface, *k*_on_ and *k*_off_ are the association and dissociation rate constant, respectively, *C*_w_ denotes the suspended antigen concentration just above the reaction surface, and *R*_T_ = 3.3 × 10^−11^ M·m is the receptor concentration.

In order to quantify the effectiveness of induce charge electrokinetics on the enhancement of the bound antigen, we define the binding enhancement factor, *Be* = *B*/*B*_0_, where *B* and *B*_0_ are the bound antigen concentration after introducing the induce charge electrokinetics at *t* = 100 s and with no applied voltages at 100 s. As for a biosensor of fixed flow rate, the binding rate is also dependent on the dimensionless parameter Damkohler number [56], *Da* = *k*_on_*R*_T_*h/D* (where, *h* is the height of the microchannel), which is the ratio of reaction velocity to diffusion velocity. The *Da* number is often used to determine whether the immuno-sensor is diffusion-limited or reaction-rate limited. When the reaction rate is faster than the diffusion of analyte to the transducer surface by convection and mass transport diffusion, the whole reaction is diffusion transport limited, in contrast, the binding rate is reaction-rate limited under the circumstance that the diffusion rate is fast and the reaction rate is slow.

### 2.4. Numerical Simulation

We performed the numerical simulation to obtain the electric field distribution, vortex flow pattern induced by ICEO slip mechanism, transportation of antigen to the receptor surface, as well as surface reaction using COMSOL Multiphysics 5.2 commercial software package (COMSOL AB, Stockholm, Sweden). Since the width of the channel can be much larger than its height, *h* = 50–100 μm, the computational domain was reduced to a 2D region of length 1 mm in the *x*-*y* plane as shown in Figure 1d. First, we acquired the two-dimensional quasi-static potential field within the fluid domain with the Laplace equation for complex potential phasor, $\nabla^{2}\phi =0$. The boundary conditions were specified potential on the driving electrode pair and zero normal voltage flux on the other insulating microchannel walls. The potential phasor of the left and right electrode were prescribed as *V*_0_ and 0, respectively. At the same time, an RC charging boundary condition, Equation (1), was imposed on the gate surface (including both electrode sidewalls for a finite electrode thickness). The ICEO slippage velocity, Equation (2), served as an effective boundary condition on the metal strip surface for the full Stokes equation, which had zero slip on other nonpolarizable channel walls due to the viscous boundary layer effect. The potential amplitude and frequency of the applied voltage signal, as well as the gate electrode height, considerably influence the ICEO convective flow pattern near the edge of floating electrode and, therefore, the bound antigen efficiency may depend on the choice of the above boundary conditions.

In the next step, the mass transport of diluted species of the antigen concentration was solved within the channel, subjected to the flux exchange condition $D\frac{\partial C}{\partial y}=\frac{\partial B}{\partial t}$ at the functionalized part of gate surface indicating a balance between the antigen binding rate at the reaction surface and diffusive dissipation rate from the neighboring bulk and zero normal flux at other non-functionalized interfaces. The initial surface concentration of bound antigen was *B* = 0 mol/m^2^ (*t* = 0), implying no binding reaction at the start, while the initial bulk concentration of freely suspended antigen was set as *C*_0_ = 10^−7^ mol/m^3^. Finally, the problem was closed with the equation describing the surface reaction kinetics Equation (4) at the functionalized part to satisfy the uniqueness theorem of the boundary value problem.

The following basic parameters were adopted in the numerical simulation: solution conductivity *σ*_f_ = 1 mS/m, immobilized antibody concentration *R*_T_ = 3.3 × 10^−11^ M·m, and the initial suspended antigen concentration *C*_0_ = 0.1 nM; the association and disassociation constant *k*_on_ and *k*_off_ for the binding reaction were 10^−6^ M^−1^·s^−1^ and 10^−3^ s^−1^, respectively, the nano-sized analyte diffusivity was *D* = 10^−11^ m^2^·s^−1^. One stationary solver was used to solve the electrostatic field amplitude and time-averaged ICEO flow field in a segregated manner, while a time-dependent solver was chosen to solve both the mass transfer within the bulk and the surface binding reaction in a fully coupled manner due to their transient nature. In addition, grid independence was checked carefully for each simulation result.

## 3. Results and Discussion

### 3.1. Concentration Simulation in Microcavity

**Figure 2:**
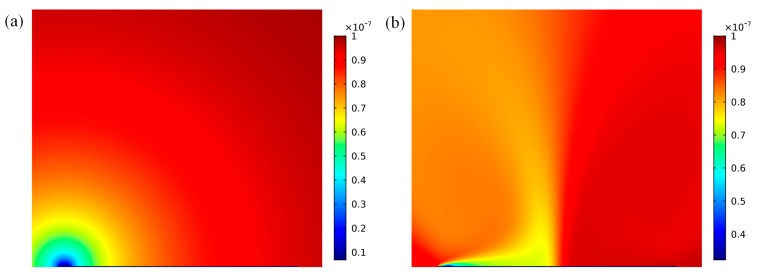
Simulation of antigen concentration distribution above the functionalized surface at 100 s for the following two cases. (**a**) Antigen concentration distribution with no external electrical field exerted, leaving mass transport diffusion on the floating surface only; (**b**) The ICEO circular flow redistributed the depleted concentration after the driving electrodes were energized with the electrical field of 4 V at 10 Hz.

As the highest flow velocities are forecasted directly on the surface of the floating electrode, we located the functionalized surface on the left side of the floating electrode surface and investigated the effect of the convective flow pattern on the binding response on top of the bipolar metal strip. Before the electrode was energized, without the effect of micro-stirring, the suspended antigen concentration depleted locally only by diffusion, as shown in Figure 2a. The mass transport limitation restrained the binding interaction between the free antigen and the immobilized receptor and lead to the growth of the diffusion boundary layer, which confined the immunoassay biosensor performance. When the driving electrode pair at two sides of the microchannel was activated at *V*_0_ = 4 V and *f* = 10 Hz, the transverse ICEO circular flow on the floating electrode redistributed the depleted concentration. As illustrated in Figure 2b, the micro-vortices over the reaction surface accelerated the fluid flow over the receptor surface, causing the efficient transport of free ligands to the functionalized surface and resulting in higher association and disassociation rates.

**Figure 3:**
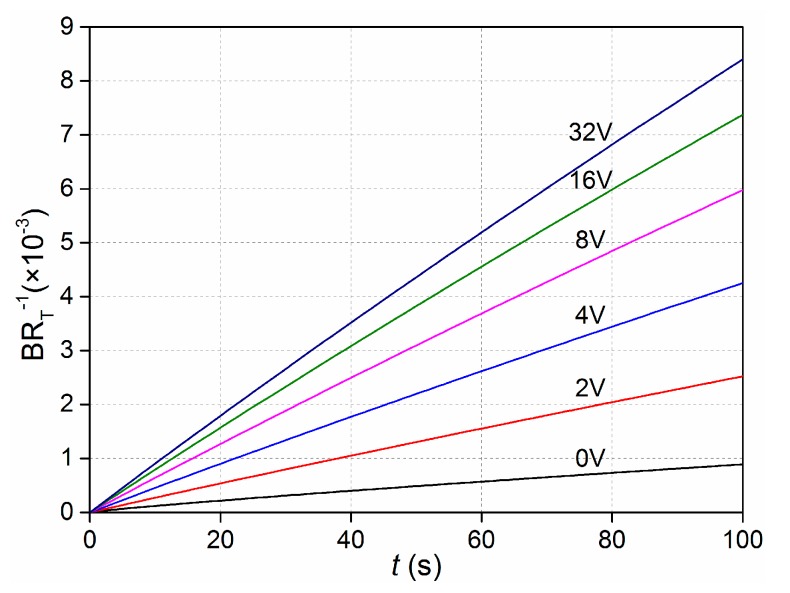
Normalized binding curves for non-enhanced (0 V) and ICEO enhanced (2 V, 4 V, 6 V, 8 V, 16 V, 32 V) transport. *B* is the amount of analyte bound, which is normalized by the concentration of immobilized antibodies, *R*_T_.

To investigate the dependence of bound antigen on applied voltage, we numerically simulated the binding rate of ICEO convective flow in an enhanced sinusoidal steady state in contrast with non-enhanced when *Da* was 660. As illustrated in Figure 3, for the binding rates, when the driving electrodes were energized with the voltage amplitude of 0 V (corresponding to the passive case), 2 V, 4 V, 8 V, 16 V, and 32 V at 100 Hz, binding rates were achieved with the increasing voltages. At low voltage, the binding ratio was proportional to the voltage magnitude as the ICEO micro-vortex enhanced the transport of antigen to the receptor surface. In addition, for a certain applied voltage, the predicted slip velocity was quadratic with the external excited signal amplitude, increasing by 4 times as the voltage doubled. As the time-averaged ICEO slip velocity on polarizable surfaces under AC forcing increases, the binding rates slow down due to the slow reaction limitation, which signifies that there exists an optimum ICEO.

**Figure 4:**
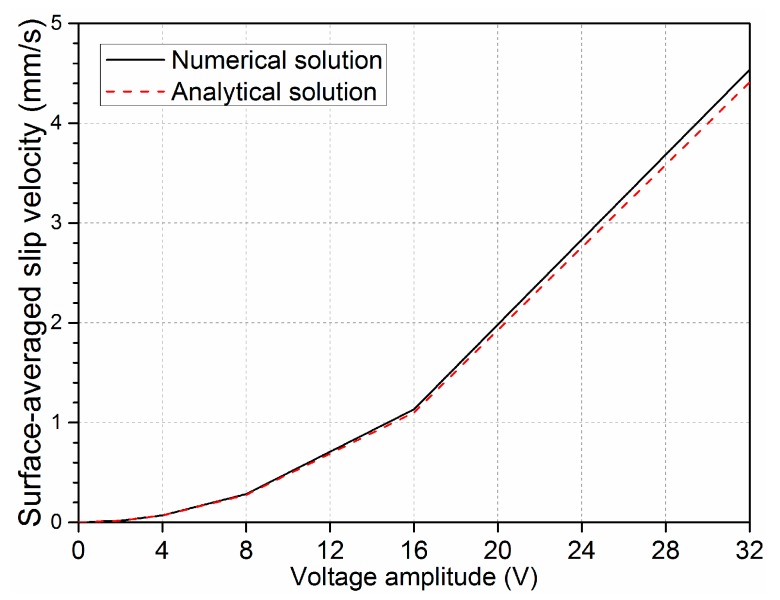
A comparison of surface-averaged slip velocity predicted by numerical and analytical solutions at different excited voltages when *Da* is 660 at 10 Hz.

When the driving electrode pair was imposed by AC voltage signal, the analytical solution for the induced zeta potential in the direct current (DC) limit can be expressed as $\tilde{\zeta }=E_{0}x/\left(1+\delta \right)$. With the simplified physical description of the double-layer dynamics, in the DC limit, the ICEO slip velocities on the electrode surface is derived with Helmholtz-Smoluchowski formula, $\langle u_{\mathrm{sDClimit}}\rangle =-\varepsilon_{f}E_{0}^{2}x/2\eta \left(1+\delta \right)$. As shown in Figure 4, the surface-averaged slip profiles calculated by analytical solution Equation (2) is in perfect coincidence with the numerical solution results at varying voltage amplitudes when *Da* is 660. Both the numerical simulation and the analytical solution can predict faster convective slip velocity on the electrode surface.

### 3.2. Dependence of the Binding Efficiency on the Damkohler Number

**Figure 5:**
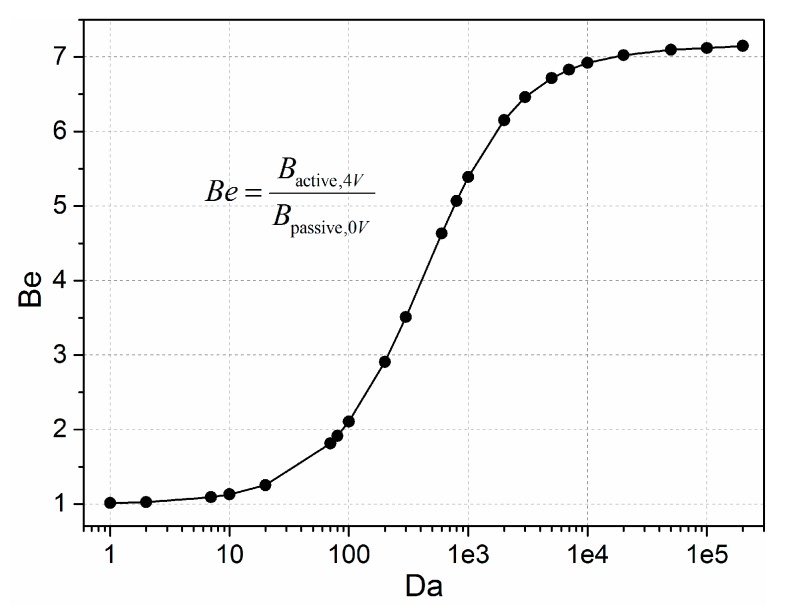
Plot for the binding enhancement factor *Be* in relation to increasing Damkohler number with the applied voltage of 4 V at 10 Hz.

In order to explore the effectiveness of ICEO circulating flow on heterogeneous immunoassays, we studied the effects of the Damkohler number upon the binding enhancement factor of the after excitation signal of 4 V at 10 Hz applied at 100 s. As depicted in Figure 5, the antigen-antibody binding efficiency as a whole enhances with the increasing *Da* number, indicating an increase in binding rate yielding a factor of 5.4 higher binding for an applied voltage of 4 V at 10 Hz when the Damkohler number is 1000. However, for smaller *Da*, the electroosmotic flow above the surface of the floating electrode cannot improve the binding rate by efficiently transporting the free antigens to the functionalized surface due to the limitation of the slow reaction. On the other hand, when *Da* is above a certain value, the reaction is too fast to leave insufficient time for association. For example, as the driving electrodes are energized by 4 V at 10 Hz, the ICEO micro-stirring is not strong enough so the binding efficiency improves only slightly when *Da* is above 10,000. Under this circumstance, the voltage signal amplitude must be further amplified to obtain larger ICEO transportation and result in a significant enhancement in binding. Based on the above discussions, we choose *Da* = 1000 for the following simulation.

### 3.3. Voltage Amplitude and Frequency of the Excitation Signal

**Figure 6:**
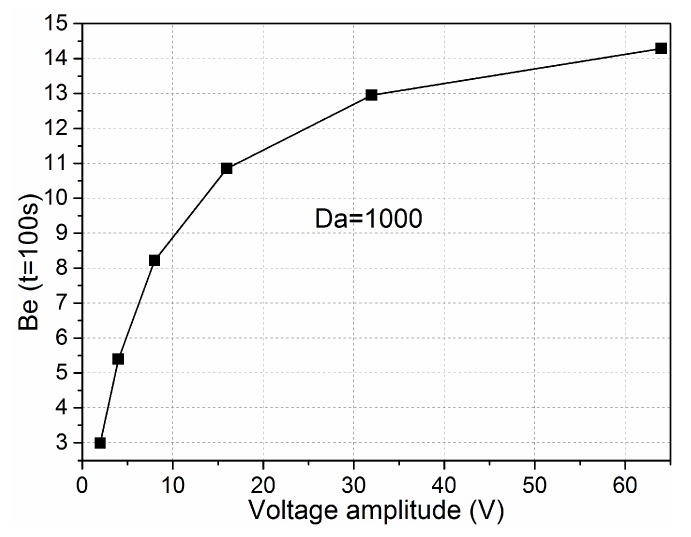
Binding enhancement factor with respect to sinusoidal signals with different voltage amplitude applied at *t* = 100 s when the *Da* = 1000.

As the ICEO recirculation motion is quadratic with voltage applied on the electrode, we investigated the relationship of binding enhancement with the voltage amplitude when the Damkohler number was 1000. As illustrated in Figure 6, when a low-frequency AC signal such as 10 Hz was applied, the binding rates increased rapidly by adjusting the gate potential of the bipolar metal strip and were proportional to the applied electrical field with the increment of voltage magnitude with voltages below 10 V. This can be explained because the reaction velocity on the floating surface is fast and the mass transport is purely diffusive in nature, which eliminates the possibility of efficient binding. In the meantime, the ICEO micro-vortex enhances the transportation of antigens to the receptor surface and improves the binding efficiency. With the increment of voltage, the binding rate strengthens slightly as the voltage is above 10 V, which is accountable for the excessively fast transversal ICEO slip on the immobilized wall compared with the limited reaction velocity. Indeed, the larger voltage is significant for the enhancement of the binding reaction, while the association and disassociation may be influenced by a too large voltage amplitude as the reaction rate cannot match with the transport velocity of analytes to the reaction surface. Under this circumstance, accelerating the ICEO convection transport by increasing the voltage amplitude may seem meaningless to the immune response.

**Figure 7:**
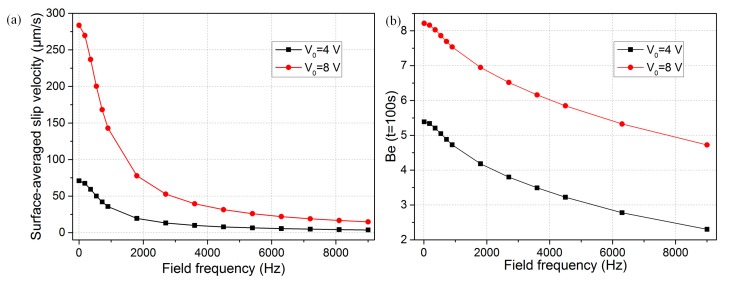
(**a**) Frequency-dependent surface-averaged ICEO slip velocity for different voltage magnitudes when *Da* is 1000; (**b**) plot of binding enhancement factor versus sinusoidal signals with different field frequencies when the voltage amplitude is 4 V and 8 V, respectively.

The optimal detecting conditions should be frequency dependent because the ICEO is very sensitive to the applied frequency of the AC voltage (Equation (2)). When the voltage signal is fixed at 4 V, the characteristic double-layer relaxation frequencies $f_{\mathrm{RC}}=\frac{\left(1+\delta \right)\sigma_{f}}{2\pi C_{D}R}$ can be obtained by carrying out simulations of varying frequencies. The ICEO slip velocities were identified at various applied frequencies. As shown in Figure 7a, the surface-averaged slip velocity decreased by 50% in the immediate vicinity of the electrode edge at *f*_RC-average_ = 900 Hz no matter whether the applied voltage was at 4 V or 8 V. In addition, the binding efficiency diminished by no more than 50% around *f*_RC_ charging frequency (Figure 7b), which can be attributed to the fact that not only convective slip velocity but also mass diffusion are involved in the immuno-reaction. In practice, we can raise the field frequency merely to 50 Hz or even 100 to 200 Hz as the binding rate declines much more slowly with field frequency compared to the ICEO slip velocity. By doing so, we can persist in enhancing the microfluidic immuno-sensors binding efficiency and simultaneously avoid the bipolar electrochemical reactions and bubble generation around the gate electrode.

### 3.4. Effect of the Gate Electrode Height

**Figure 8:**
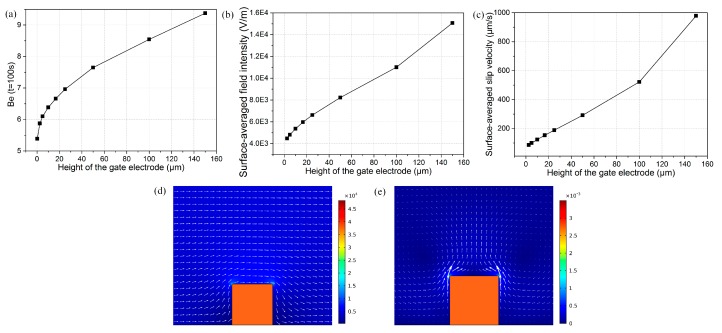
At *f* = 10 Hz and *V*_1_ = 4 V, plot of: (**a**) bound antigen enhancement vs. height of gate electrode; (**b**) surface-averaged field intensity predicted at different gate electrode heights; (**c**) height-dependent surface-averaged ICEO velocityies on top of the bipolar electrode; (**d**) and (**e**) surface and arrow plots around the surface of floating electrode: (**d**) electric field distribution around the bipolar metal strip; (**e**) ICEO fluid flow field around metal surface.

We now explore the effect of gate electrode height on binding enhancement at the signal frequency of 10 Hz and potential amplitude of 4 V. Figure 8a demonstrates the relationship of bound antigen enhancement factor vs. electrode height after the driving electrode pair was energized at 100 s. It can be seen that the binding rate strengthened with the increasing gate electrode height, which may be attributed to the following three reasons: (1) Since this is the low-frequency limit, the electric field lines resembles that of an insulator just outside the induced double layer of the gate electrode. In the situation of a flat metal surface immersed in electrolyte, the bulk electric field is tangential and uniform just outside the diffuse screening cloud from the viewpoint of an observer. However, with increasing electrode height, the electric field bends around the surface of the “insulating” conductor, due to complete double-layer polarization, and becomes unevenly distributed. This geometric change increases the field intensity just at the electrode corner where the radius of curvature is largest, rendering an intensified surface-averaged tangential field outside the Debye layer, as shown in Figure 8b. Since electroosmotic flow is proportional to the magnitude of the tangential field that is augmented with electrode height, the nonlinear ICEO slip velocity becomes larger for a higher gate electrode (Figure 8c), giving rise to more prominent convective transportation of antigen to the functionalized surface; (2) There exists electroosmotic slips at both sidewalls of the metal strip for 3D electrode structure (Figure 8e), but those disappear for a flat shape (Figure 1f), so enlargement of the effective slip area accelerates the convective mass transport at a large aspect ratio as well; (3) Since the bulk flow field on both sides of the top surface of the 3D gate electrode can now be driven by electroosmotic slip, as compared to the no-slip walls in the flat configuration which permits no fluid motion, the effect of the viscous boundary layer on the surface ICEO slippage diminished, enhancing the convective transport and improving the surface bound immuno-reaction.

### 3.5. Binding Ehancement in a Continuous Base Flow

**Figure 9:**
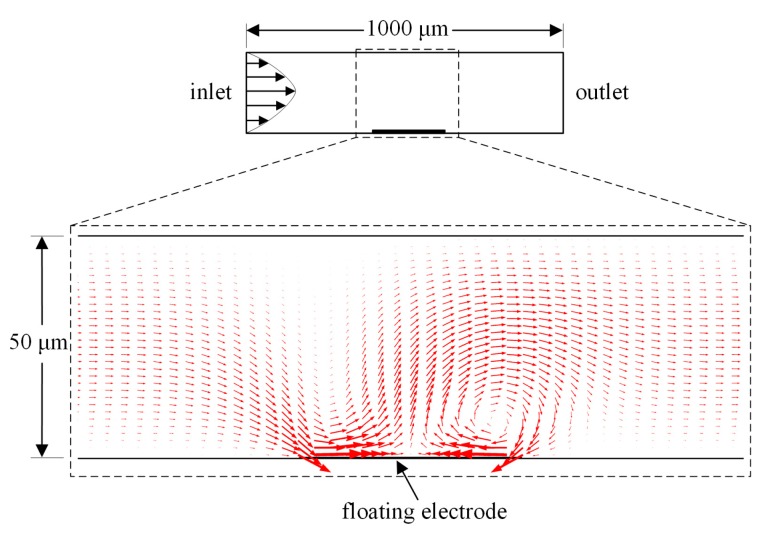
Velocity field distribution in the microchannel subjected to ICEO slippage on the bipolar metal strip when the driving electrode pair was energized with an applied external electrical field magnitude of 8 V. The inlet mean velocity for the parabolic pressure-driven flow was 50 μm·s^−1^. The schematic is not to scale.

To address a more realistic situation in a microdevice which needs continuous sample handing, we have to examine the effectiveness of the ICEO convective flow above the gate electrode surface in a microchannel with axial pressure-driven sample injection on the binding reaction. To do this, we defined the boundary condition at the inlet plane with a parabolic velocity profile of a mean velocity *u*_0_ = 50 μm·s^−1^ for the numerical simulation. Compared with 200 μm in the above discussion, we lowered the microchannel height to 50 μm (Figure 9) to better depict the effect of ICEO surface slippage on the antigen-antibody bound reaction under the influence of a continuous pressure-driven flow, since the binding enhancement factor *Be* is not expected to be as high as that in the previous static flow condition. With the driving voltage at 8 V, the ICEO velocity produced above the blocking electrode was about 200 μm·s^−1^, which was large compared with the average base flow.

**Figure 10:**
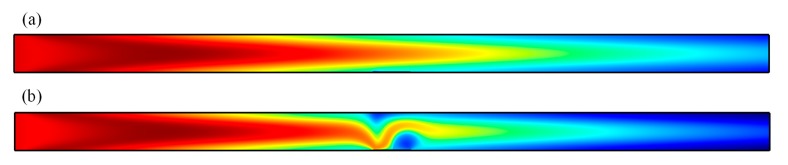
Concentration distribution of ICEO modified channel flow at *t* = 17 s when the driving electrode pair was energized with voltage signals of (**a**) 0 V; and (**b**) 8 V. Here, the red, blue, and green colors represent the different rates of binding concentration to initial concentration, namely, 1, 0.5, and 0, respectively.

As shown in Figure 10, with the pressure-driven flow continuously introduced into the microchannel and no voltage applied, the base flow is parabolic; therefore, the replenishment of consumed analyte near the binding surface solely depends on the axial convective mass transport and transverse diffusive flux. When the driving electrode pair is activated, the concentration distribution is distorted by ICEO convective flow, providing more chance for association and disassociation between antigen and antibody. Therefore, the ICEO micro-vortex is efficient in transporting the free ligands to the immobilized functionalized reaction surface and refreshing the consumed analyte near the metal strip surface as indicated by the large concentration gradient above the gate, resulting in enhanced reaction kinetics.

**Figure 11:**
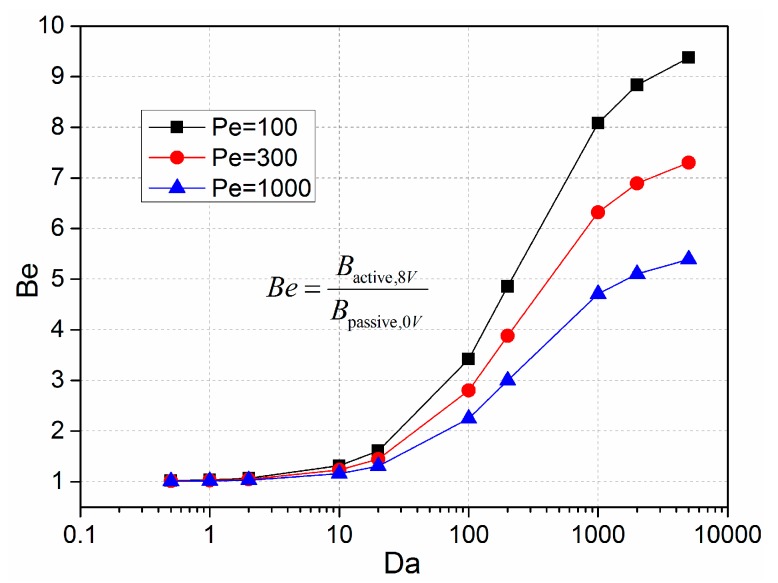
Binding enhancement factor *Be* with regard to the increasing Damkohler number at varying Peclet numbers *Pe*. The Peclet number is varied through changing the inlet flow velocity, while the *Be* factor is adjusted by varying the value of *k*_on_.

We introduced the ratio of convective to diffusive transport, Peclet number *Pe = uh/D*, to describe the interrelationships between inlet flow velocity and the bound reaction. Figure 11 presents the binding enhancement factor *Be* for varying *Da* numbers with different Pelect numbers, namely, 100, 300, and 1000. It can be seen that the binding enhancement factor increases with the *Da* number for a certain inlet flow velocity, which is in good accordance with the results discussed in Section 3.2. In addition, the higher inlet flow velocity can enhance the longitudinal mass transport of the analyte, however, the enhancement factor decreases with the increasing Peclet number. This means that the ICEO convective flow is less efficient for higher inlet flow velocities due to the lower probability of the bound interaction between the free antigen with higher axial velocity and the immobilized antibody on the reaction surface.

## 4. Conclusions

In summary, we investigated the ICEO effect on the binding enhancement of antigens on a functionalized surface by performing a 2D simulation on heterogeneous immunoassays in a microchannel with a floating gate electrode deposited on the channel bottom. The ICEO micro-vortex significantly increased the reaction rate to accelerate both the association and dissociation processes. The dependence of enhancement on voltage magnitude, field frequency, and floating electrode height was analyzed. It was demonstrated that the detection sensitivity and limit of detection were improved due to the ICEO micro-vortices on the surface of the floating electrode. Overall, the proposed rapid and label-free platform possesses great potential in the point-of-care diagnostics field.

## References

[1] Chen P., Chung M.T., McHugh W., Nidetz R., Li Y., Fu J., Cornell T.T., Shanley T.P., Kurabayashi K. (2015). Multiplex serum cytokine immunoassay using nanoplasmonic biosensor microarrays. ACS Nano.

[2] Asensio L., González I., García T., Martín R. (2008). Determination of food authenticity by enzyme-linked immunosorbent assay (ELISA). Food Control.

[3] Chen X., Wang Y., Zhou J., Yan W., Li X., Zhu J.-J. (2008). Electrochemical impedance immunosensor based on three-dimensionally ordered macroporous gold film. Anal. Chem..

[4] Abdulhalim I., Zourob M., Lakhtakia A. (2008). Surface plasmon resonance for biosensing: A mini-review. Electromagnetics.

[5] Deng X., Chen M., Fu Q., Smeets N.M., Xu F., Zhang Z., Filipe C.D., Hoare T. (2016). A highly sensitive immunosorbent assay based on biotinylated graphene oxide and the quartz crystal microbalance. ACS Appl. Mater. Interfaces.

[6] Ng A.H.C., Uddayasankar U., Wheeler A.R. (2010). Immunoassays in microfluidic systems. Anal. Bioanal. Chem..

[7] Selmi M., Echouchene F., Gazzah M.H., Belmabrouk H. (2015). Flow confinement enhancement of heterogeneous immunoassays in microfluidics. IEEE Sens. J..

[8] Shen Y., Song Z., Yan Y., Song Y., Pan X., Wang Q. (2017). Automatic and selective single cell manipulation in a pressure-driven microfluidic lab-on-chip device. Micromachines.

[9] Han D., Park J.K. (2016). Optoelectrofluidic enhanced immunoreaction based on optically-induced dynamic AC electroosmosis. Lab Chip.

[10] Hart R., Lec R., Noh H.M. (2010). Enhancement of heterogeneous immunoassays using AC electroosmosis. Sens. Actuators B.

[11] Liu X., Yang K., Wadhwa A., Eda S., Li S., Wu J. (2011). Development of an AC electrokinetics-based immunoassay system for on-site serodiagnosis of infectious diseases. Sens. Actuators A.

[12] Hu G., Gao Y., Sherman P.M., Li D. (2005). A microfluidic chip for heterogeneous immunoassay using electrokinetical control. Microfluid. Nanofluid..

[13] Hu G., Gao Y., Li D. (2007). Modeling micropatterned antigen-antibody binding kinetics in a microfluidic chip. Biosens. Bioelectron..

[14] Hyoung Kang K., Xuan X., Kang Y., Li D. (2006). Effects of DC-dielectrophoretic force on particle trajectories in microchannels. J. Appl. Phys..

[15] Ren Y., Ao H., Gu J. (2009). Research of micro-particle manipulation in micro systems using DEP force. Acta Phys. Sin..

[16] Harrison H., Lu X., Patel S., Thomas C., Todd A., Johnson M., Raval Y., Tzeng T.-R., Song Y., Wang J. (2015). Electrokinetic preconcentration of particles and cells in microfluidic reservoirs. Analyst.

[17] Zhu L., Patel S.H., Johnson M., Kale A., Raval Y., Tzeng T.-R., Xuan X. (2016). Enhanced throughput for electrokinetic manipulation of particles and cells in a stacked microfluidic device. Micromachines.

[18] Do J., Ahn C.H. (2008). A polymer lab-on-a-chip for magnetic immunoassay with on-chip sampling and detection capabilities. Lab Chip.

[19] Hofmann O., Voirin G., Niedermann P., Manz A. (2002). Three-dimensional microfluidic confinement for efficient sample delivery to biosensor surfaces. Application to immunoassays on planar optical waveguides. Anal. Chem..

[20] He J.-L., Wang D.-S., Fan S.-K. (2016). Opto-microfluidic immunosensors: From colorimetric to plasmonic. Micromachines.

[21] Ramos A., Morgan H., Green N.G., Castellanos A. (1998). AC electrokinetics: A review of forces in microelectrode structures. J. Phys. D.

[22] Zhao C., Ge Z., Yang C. (2017). Microfluidic techniques for analytes concentration. Micromachines.

[23] Mavrogiannis N., Desmond M., Ling K., Fu X., Gagnon Z. (2016). Microfluidic mixing and analog on-chip concentration control using fluidic dielectrophoresis. Micromachines.

[24] Jia Y., Ren Y., Jiang H. (2015). Continuous dielectrophoretic particle separation using a microfluidic device with 3D electrodes and vaulted obstacles. Electrophoresis.

[25] Zhu J., Xuan X. (2009). Dielectrophoretic focusing of particles in a microchannel constriction using DC-biased AC flectric fields. Electrophoresis.

[26] Xie C., Chen B., Wu J. (2017). Three-dimensional interaction of a large number of dense DEP particles on a plane perpendicular to an AC electrical field. Micromachines.

[27] Xuan X., Li D. (2005). Electroosmotic flow in microchannels with arbitrary geometry and arbitrary distribution of wall charge. J. Colloid Interface Sci..

[28] Li F., Jian Y., Xie Z., Liu Y., Liu Q. (2017). Transient alternating current electroosmotic flow of a jeffrey fluid through a polyelectrolyte-grafted nanochannel. RSC Adv..

[29] Chang L., Jian Y., Buren M., Sun Y. (2016). Electroosmotic flow through a microparallel channel with 3D wall roughness. Electrophoresis.

[30] Bashirzadeh Y., Maruthamuthu V., Qian S. (2016). Electrokinetic phenomena in pencil lead-based microfluidics. Micromachines.

[31] Li S., Ren Y., Cui H., Yuan Q., Wu J., Eda S., Jiang H. (2015). Alternating current electrokinetics enhanced in situ capacitive immunoassay. Electrophoresis.

[32] Li S., Ren Y., Jiang H. (2014). Convection and mass transfer enhanced rapid capacitive serum immunoassay. RSC Adv..

[33] Wu Y., Ren Y., Tao Y., Hou L., Hu Q., Jiang H. (2017). A novel micromixer based on the alternating current-flow field effect transistor. Lab Chip.

[34] Jiang H.Y., Ren Y.K., Ao H.R., Antonio R. (2008). Electrohydromechanical analysis based on conductivity gradient in microchannel. Chin. Phys. B.

[35] Liu W., Shao J., Ren Y., Liu J., Tao Y., Jiang H., Ding Y. (2016). On utilizing alternating current-flow field effect transistor for flexibly manipulating particles in microfluidics and nanofluidics. Biomicrofluidics.

[36] Sigurdson M., Wang D., Meinhart C.D. (2005). Electrothermal stirring for heterogeneous immunoassays. Lab Chip.

[37] Feldman H.C., Sigurdson M., Meinhart C.D. (2007). AC electrothermal enhancement of heterogeneous assays in microfluidics. Lab Chip.

[38] Sugioka H. (2008). Suppression of reverse flows in pumping by induced-charge electro-osmosis using asymmetrically stacked elliptical metal posts. Phys. Rev. E.

[39] Paustian J.S., Pascall A.J., Wilson N.M., Squires T.M. (2014). Induced charge electroosmosis micropumps using arrays of janus micropillars. Lab Chip.

[40] Harnett C.K., Templeton J., Dunphy-Guzman K.A., Senousy Y.M., Kanouff M.P. (2008). Model based design of a microfluidic mixer driven by induced charge electroosmosis. Lab Chip.

[41] Wu Z., Li D. (2008). Micromixing using induced-charge electrokinetic flow. Electrochim. Acta.

[42] Daghighi Y., Li D. (2013). Numerical study of a novel induced-charge electrokinetic micro-mixer. Anal. Chim. Acta.

[43] Prabhakaran R.A., Zhou Y., Zhao C., Hu G., Song Y., Wang J., Yang C., Xuan X. (2017). Induced charge effects on electrokinetic entry flow. Phys. Fluids.

[44] Squires T.M., Bazant M.Z. (2004). Induced-charge electro-osmosis. J. Fluid Mech..

[45] Bazant M.Z., Squires T.M. (2004). Induced-charge electrokinetic phenomena: Theory and microfluidic applications. Phys. Rev. Lett..

[46] Bazant M.Z., Squires T.M. (2010). Induced-charge electrokinetic phenomena. Curr. Opin. Colloid Interface Sci..

[47] Ren Y., Liu W., Jia Y., Tao Y., Shao J., Ding Y., Jiang H. (2015). Induced-charge electroosmotic trapping of particles. Lab Chip.

[48] Ren Y., Liu J., Liu W., Lang Q., Tao Y., Hu Q., Hou L., Jiang H. (2016). Scaled particle focusing in a microfluidic device with asymmetric electrodes utilizing induced-charge electroosmosis. Lab Chip.

[49] Song Y., Wang C., Li M., Pan X., Li D. (2016). Focusing particles by induced charge electrokinetic flow in a microchannel. Electrophoresis.

[50] Tao Y., Ren Y., Liu W., Wu Y., Jia Y., Lang Q., Jiang H. (2016). Enhanced particle trapping performance of induced charge electroosmosis. Electrophoresis.

[51] Wu Y., Ren Y., Tao Y., Hou L., Jiang H. (2016). Large-scale single particle and cell trapping based on rotating electric field induced-charge electroosmosis. Anal. Chem..

[52] Ren Y., Liu W., Liu J., Tao Y., Guo Y., Jiang H. (2016). Particle rotational trapping on a floating electrode by rotating induced-charge electroosmosis. Biomicrofluidics.

[53] Pascall A.J., Squires T.M. (2010). An automated, high-throughput experimental system for induced charge electrokinetics. Lab Chip.

[54] Myszka D.G., He X., Dembo M., Morton T.A., Goldstein B. (1998). Extending the range of rate constants available from biacore: Interpreting mass transport-influenced binding data. Biophys. J..

[55] Hibbert D.B., Gooding J.J., Erokhin P. (2002). Kinetics of irreversible adsorption with diffusion: Application to biomolecule immobilization. Langmuir.

[56] Deen W.M. (1998). Analysis of Transport Phenomena, Topics in Chemical Engineering.

